# DNA Methylation and Histone Modification in Hypertension

**DOI:** 10.3390/ijms19041174

**Published:** 2018-04-12

**Authors:** Shaunrick Stoll, Charles Wang, Hongyu Qiu

**Affiliations:** 1 Division of Pharmacology and Physiology, Department of Basic Sciences, School of Medicine, Loma Linda University, Loma Linda, CA 92350, USA, sstoll@llu.edu; 2 Center for Genomics, Department of Basic Sciences, School of Medicine, Loma Linda University, Loma Linda, CA 92350, USA; chwang@llu.edu

**Keywords:** DNA methylation, histone modifications, vascular smooth muscle cells, endothelial cells, hypertension

## Abstract

Systemic hypertension, which eventually results in heart failure, renal failure or stroke, is a common chronic human disorder that particularly affects elders. Although many signaling pathways involved in the development of hypertension have been reported over the past decades, which has led to the implementation of a wide variety of anti-hypertensive therapies, one half of all hypertensive patients still do not have their blood pressure controlled. The frontier in understanding the molecular mechanisms underlying hypertension has now advanced to the level of epigenomics. Particularly, increasing evidence is emerging that DNA methylation and histone modifications play an important role in gene regulation and are involved in alteration of the phenotype and function of vascular cells in response to environmental stresses. This review seeks to highlight the recent advances in our knowledge of the epigenetic regulations and mechanisms of hypertension, focusing on the role of DNA methylation and histone modification in the vascular wall. A better understanding of the epigenomic regulation in the hypertensive vessel may lead to the identification of novel target molecules that, in turn, may lead to novel drug discoveries for the treatment of hypertension.

## 1. Introduction

Systemic hypertension (or Hypertension in this review) refers to a condition of high blood pressure (BP) in the systemic arteries, the vessels that carry blood from the heart to the body’s tissues, which distinguishes the condition from a local high BP such as in pulmonary (lung) hypertension. Hypertension has been defined as a BP reading of 140/90 mm Hg or higher in adults for many years. However, the latest report (2017) from the American College of Cardiology/American Heart Association (ACC/AHA) provides an updated guideline that classifies hypertension as a BP reading of 130/80 mm Hg or higher [1]. Hypertension remains a major risk factor for myocardial infarction, heart failure, end-stage renal disease, and stroke despite extensive research for decades and many therapeutic approaches [2]. Hypertension affects nearly one third of all adults in the US and only one half of the hypertensive patients have their BP under control [3]. The ACC/AHA report also notes that males are more likely to develop hypertension compared to women at the pre-menopausal ages, suggesting a role for hormone signaling in the management of BP [1]. Although the causes of hypertension have not been fully outlined, certain chronic conditions may increase the risk of the development of hypertension which include general risk factors such as aging, smoking, low socioeconomic and educational status, overweight/obesity, unhealthy diet, and physical inactivity as well as other secondary disorders such as chronic kidney disease (CKD), genetic family history, diabetes mellitus, obstructive sleep apnea and psychosocial stress [1]. Current nonpharmacological therapy focuses on maintaining a healthy diet, exercise, and other lifestyle changes, while pharmacological treatment includes: thiazide or thiazide-type diuretics, angiotensin-converting enzyme (ACE) inhibitors, dihydropyridines, aldosterone receptor blockers (spironolactone and derivatives), beta blockers, and vasodilators, direct renin inhibitors, and alpha-1 blockers [1]. Despite the plethora of treatment options, the high BP of 16.1 million hypertensive patients remains uncontrolled [3].

While many signaling pathways involved in the development of hypertension have been discovered, the molecular mechanisms related to the epigenomic regulation underlying vascular dysfunction are still not well understood. Epigenomics refers to the genome-wide study of gene regulation that alters gene activity without changing the DNA sequence [2]. Epigenomic regulation provides a link between the genotype and phenotype and is essential for many normal cellular functions, and inappropriate regulation at the epigenomic level may lead to some major adverse effects on cellular function of a tissue or organ which may lead to the development of diseases. Different epigenetic regulations have been identified in hypertension, including methylation, acetylation, phosphorylation, ubiquitination, and sumolyation.

It has been well-known that the renin-angiotensin-aldosterone system (RAAS), a hormone system that is integral to the physiological regulation of BP, plays a crucial role in the development of hypertension, thus, the epigenomic alterations of RAAS-regulated genes and their effects have been extensively tested in hypertensive models [4]. Increasing evidence is emerging that epigenomics also plays an important role in gene expression which may alter the phenotype and function of the vascular wall or cells in response to environmental stresses [5], underscoring our need to consider the contribution of epigenomic regulation of the vascular walls to hypertension. This review seeks to highlight recent advances in our knowledge of the changes in DNA methylation and histone modification which occur during the development of hypertension, as summarized in Table 1. Despite the well-known importance of RAAS in the development of hypertension, we have only briefly summarized them in this present manuscript since the relative epigenomic alterations of RAAS have been extensively discussed in a recent review [4]. To concentrate on the updated information, our present review focuses on the contribution of alterations to the vasculature in the development of systemic hypertension. An increased understanding of the epigenomic regulation of these vascular cells will lead to the identification of novel target molecules that may, in turn, lead to novel drug discoveries for the treatment of hypertension.

## 2. Discovery, Development, and Detection

### 2.1. DNA Methylation

DNA methylation involves the addition of methyl groups to the cytosine residues of DNA [24]. Initial studies of X-chromosome inactivation in mice provided the evidence that DNA could be silenced without changes in the DNA sequence itself [25,26]. Later studies showed that DNA methylation can act as a mechanism through which this silencing may occur [27,28]. Importantly, DNA methylation marks can be copied from the parent strand to the daughter strand. Another notable finding is the identification of the main methylation target: the sequence of CpG, shorthand for 5′-C-phosphate-G-3′, that is, cytosine and guanine separated by only one phosphate group [27,28]. These findings eventually led to the development of methylation-sensitive restriction enzymes, which became important tools in epigenetic analyses [29,30]. 

DNA methlytransferases are responsible for moving methyl groups from S-adenosyl methionine (SAM) to CpG islands on DNA. For example, DNA methlytransferases 1, 3A, and 3B (DNMT1/3A/3B) add methyl groups to the carbon at position 5 of cytosine resides which are adjacent to guanine residues to produce 5-methylcytosine (5mC) [31,32]. While CpG is the main target, methylation may occur on CpHpG, where H may be A, T, or C [31]. The methylation mark 5mC is generally associated with gene repression within the gene promoter [31,33]. Alternatively, actively transcribed genes may be methylated within the transcriptional region which includes parts actually translated into protein (exons) and parts that will be cut out of the mRNA and are not translated into protein (introns) [31]. The methylation pattern of a cell may vary in response to stress, as different genes are turned on and off. For example, it was shown that in the nuclei of mouse cardiomyocytes, 127 genes gained methylation and 313 lost methylation of their transcriptional region during the postnatal period [34]. While methylation at transcription start sites represses transcription, it is yet unclear whether methylation within exons serve clear functions, as high methylation seems to be positively correlated with transcription. One suggestion is that DNA methylation within exons may affect alternative splicing [35].

Methylation patterns can also change under stress and disease, as shown in the methylation of promoters of tumor suppressor genes in cancer [36]. Importantly, DNA methylation is reversible, with the removal reaction catalyzed by histone lysine demethylases (KDM) [37], providing therapeutic potential to prevent or change the pathological DNA methylation that may be related to the diseases.

Initial methylation detection assays utilized restriction endonucleases with known methylation sensitive CpG sites and their methylation insensitive isoschizomers [30]. Genomic DNA was then subjected to cleavage by both sets of restriction enzymes, and the differences between the fragments would outline where the methylated cytosines were, as revealed by Southern blot analysis, 2-dimensional gel electrophoresis, polymerase chain reaction (PCR), and, more recently, next-generation sequencing [31]. Alternatively, methylated cytosines may be targeted using specific antibodies by a process known as methylated DNA immunoprecipitation (MeDIP) or affinity enrichment [38]. One of the most common current methods of detecting epigenetic modifications is bisulfite sequencing. Unmethylated cytosine residues are converted to uracil using sodium bisulfite and alkaline treatment, while methylated cytosines are left intact. The bisulfite-treated DNA is then sequenced to reveal the methylated cytosines [31].

In addition to DNA methylation (5mC), other DNA modifications include hydroxymethylation (5hmC) and formylcytosine (5fC) [39]. Ten eleven translocation (TET) enzymes oxidize 5mCs to 5hmCs, and further catalyze the conversion to 5fC and 5-carboxylcytosine (5-caC) which can then be replaced by unmethylated cytosine after undergoing thymine DNA glycosylase (TDG)-mediated base excision and DNA base excision repair [33,40]. The TET enzymes may be significant to future therapies as they allow exploitation of the oxidation of 5mCs as a means of reversing gene repression by DNA methylation, should the repression of signature genes be identified.

### 2.2. Histone Modification

Histones are important proteins responsible for maintaining the structure of chromatin and play a role in the dynamic and long term regulation of genes. The *N*-terminal tail of histone 3 (H3), one of the five histones found in eukaryotic nuclei, is subject to methylation or acetylation of lysine and arginine residues as well as phosphorylation of serine and threonine residues [41]. Histones are acetylated by histone acetyltransferases (HAT) and deacytelated by histone deacetylases (HDAC). These modifications may have opposing effects based on which residue is modified or which moiety is added. For example, methylation of H3 lysine 4 (H3K4) is a hallmark of actively transcribed DNA while methylation of H3 lysine 9 (H3K9) is associated with repressed gene expression [42,43]. Acetylation of histones is associated with transcriptionally active DNA, as the *N*-terminal of the histone tails become neutralized and their affinity for DNA reduced, so loosening the conformation of the chromatin [44]. Interestingly, while methylation at H3K9 allows for the binding of heterochromodoman protein 1 (HP1), leading to the repression of transcription, methylation at H3K4 blocks the binding of transcriptional repressor, nucleosome remodeling and deacetylase (NuRD), leading to transcription. Acetylation of lysines allows for the binding of bromodomain proteins, such as histone acetyltransferase, GCN5 [43].

The most common histone modification assay is the chromatin immunoprecipitation (ChIP), which uses antibodies against site-specific epigenetic marks to identify histone-DNA complexes with that mark [45]. Following fragmentation, histone-DNA complexes that showcase specific modifications are immunoprecipated using an antibody against that modification. Enriched and purified DNA fragments can then be detected by quantitative PCR (ChIP-qPCR), microarray analysis (ChIP-on-chip) or deep sequencing (ChIP-seq) [46]. ChIP-seq uses next-generation sequencing instead of amplifying purified DNA that was hybridized to a DNA microarray [47]. Importantly, ChIP-seq allows for mapping of the newly sequenced DNA to the reference genome, enabling researchers to determine the genome-wide distribution of particular modifications [46].

Conventional epigenomic analyses require large amounts of starting material due to the low DNA recovery efficiency after bisulfite conversion and enzyme digestion [48]. However, using large sample sizes masks cell to cell heterogeneity which may be important to the disease state. As molecular technology progresses to the single-cell level, many new approaches are being developed to study the single-cell epigenome, albeit with limited efficiency and scalability [39,48], including: single-cell bisulfite sequencing [49], single-cell reduced representation bisulfite sequencing [50], DNase I sequencing [51], single-cell DamID sequencing [52] and single-cell ATAC-seq [53]. Drawbacks of these methods include low coverage of CpG islands as compared to bulk methods, but the methodology is expected to grow to meet these challenges [54]. One such recent development is single cell combinatorial indexing for methylation analysis (sci-MET) which boasts of a 69% alignment rate to bulk methods [55].

## 3. Epigenomic Regulation in Hypertensive Vasculature

While numerous studies have outlined the epigenetic mechanisms in pulmonary hypertension (reviewed in [56,57,58,59]), the epigenomic regulation in systemic hypertension remains largely undescribed. Due to the well-known involvement of the renin-angiotensin-aldosterone system (RAAS) system on arterial pressure regulation, the effects of epigenomic regulation of the RAAS system have been extensively tested in animal models of systemic hypertension [4]. For example, it has been shown that the angiotensin 1α receptor (AT1aR), encoded by *Atgr1α*, is significantly increased in spontaneously hypertensive rats (SHR) compared to its counterpart, the Wistar-Kyoto rats (WKY), which may be responsible for the increased BP in SHR [6]. Bisulfite sequencing further revealed that the *Atgr1*α promoter is hypomethylated at its CpG islands in SHR at 20 weeks compared to WKY rats, implicating that the methylation of *Atgr1α* CpG islands reduces its expression, leading to normotensive BP [6]. In addition, hypomethylation of the promoter regions of the angiotensin II type 1β receptor (AT1bR) gene, (*Atgr1β*), in the adrenal glands of the maternal low protein rat exhibited hypertension in response to salt intake [7]. A similar effect was seen in another study in mice where maternal protein deficiency during pregnancy reduced methylation of promoter regions of the angiotensin I converting enzyme gene (*Ace-1*), which is responsible for converting angiotensin I to the active angiotensin II, eventually leading to hypertension in offspring [8]. In human cell lines, the luciferase activity of the *ACE-1* promoter/reporter constructs of somatic ACE (sACE) was inhibited by DNA methylation and subsequent inhibition of DNA methylation and/or histone deacetylation by 5-aza-cytidine injections in rats restored sACE expression in the lung and liver, highlighting the epigenetic regulation of sACE in hypertension [9]. 

The hydroxysteroid dehydrogenase-11β2 enzyme (HSD11B2) is responsible for degrading cortisol to biologically inert cortisone. Cortisol can be found in the blood at concentrations 2-3 orders of magnitude higher than aldosterone, the key mineralocorticoid in the RAAS [60]. Although cortisol and aldosterone bind mineralocorticoid receptors with similar affinity, degradation of cortisol to cortisone by the HSD11B2 enzyme in mineralocorticoid target tissues ensures that aldosterone is able to bind to the mineralocorticoid receptors [61]. In normal situations, aldosterone regulates sodium reabsorption due to the inactivation of cortisol to cortisone by the 11β HSD enzyme. Thus cortisol only has effects in the absence of this enzyme. Hypermethylation of the *HSD11B2* gene promoter impairs HSD11B2-mediated degradation of cortisol to cortisone, leading to an altered tetrahydrocortisol (THF) to tetrahydrocortisone (THE) ratio [10,62]. A high concentration of cortisol in mineralocorticoid target tissues and enables cortisol to regulate sodium reabsorption by the kidney, and ultimately arterial pressure. The hypermethylation of the HSD11B2 promoter may also contribute to the development of apparent mineralocorticoid excess (AME) [63]. This presents a case where both genetic and epigenetic regulation may be present.

Interestingly, in addition to the reports of the regulation of DNA methylation on RAAS, SHR aortas also showed higher enrichment of H3Ac and H3K4me3, while enrichment of H3K9me2 was reduced on the angiotensin-converting enzyme 1 (*ACE1*) promoter [19]. Thus, both the regulation of DNA methylation and histone modification exist on RAAS system.

Despite the importance of epigenetic regulation in the RAAS system on arterial pressure regulation, the blood vessels are the end-effect targets of RAAS and the function of blood vessels, particular the arteries, play a determinant role in the control of BP. Thus, in addition to the epigenetic regulation in the RAAS system described above, we will outline the recent studies on the epigenomic regulation in vascular tissue and cells, focusing on DNA methylation and histone modification in hypertension.

### 3.1. Epigenomic Regulation in the Whole Vessel

Several epigenomic studies have been conducted at the level of the whole vessel in animals. The Na^+^–K^+^–2Cl^−^ cotransporter 1 (NKCC1), which is encoded by *Slc12a2*, regulates the exchange of sodium, potassium, and chlorine ions across cells of various types, including VSMCs and endothelial cells, regulating ionic balance and cell volume [64]. It has been showed that NKCC1^−/−^ mice displayed a >15 mmHg reduction in systolic BP compared to wild-type and reduced ability to maintain vascular tone [65]. Pharmacological inhibition of NKCC1 by bumetanide led to an immediate 5% reduction in BP, highlighting its importance in BP regulation [66]. Combined bisulfite restriction assay and bisulfite sequencing revealed that the *Slc12a2* promoter was hypomethylated in SHR aorta and heart compared to WKY, which resulted in an upregulation of NKCC1 in both mRNA and protein levels detected by PCR and western blots in SHR vs WKY [11].

Furthermore, DNA methylation has been also linked to the estrogen receptor-mediated vascular regulation. Estrogen induces vasodilation as well as inhibits the response of blood vessels to injury, by interacting directly with the vasculature [67]. Two estrogen receptors have been identified: estrogen receptor α (ERα) and estrogen receptor β (ERβ). In uterine arteries of pregnant sheep, hypermethylation of the ERα (*ESR1*) promoter during hypoxia reduced its expression, leading to preeclampsia and impaired cardiovascular homeostasis [12]. Promoter hypermethylation inhibited transcription factor binding and promoter activity, leading to gene repression [12]. Although these mechanisms need to be confirmed in the human, this evidence suggests that the protective nature of estrogen signaling may be regulated through epigenetic mechanisms, which may also contribute to the gender disparity in hypertensive patients.

Moreover, histone modification has been found to be involved in the regulation of vascular function in hypertension. Additionally, NKCC1 can also be regulated by histone modification. Angiotensin II (Ang II) was delivered in rats, in vivo, to increase BP and the changes in NKCC1 mRNA, protein and epigenetic modifications at the *slc12a2* promoter were measured in the aorta [23]. Real-time PCR and western blot revealed a progressive increase in NKCC1 expression over the period of Ang II delivery. Interestingly, H3Ac levels were consistently increased in Ang II infused rats whereas H3K27me3 (a repressive histone code) levels were decreased as compared to sham [23]. These results together suggest that NKCC1 might be regulated by both DNA methylation and histone modification [4].

Overexpression of SIRT1, a histone deacetylase, reduced angiotensin-II induced hypertrophy, in vitro, and vascular remodeling and hypertension, in vivo [68,69]. Among the affected parameters were reduced reactive oxygen species (ROS) generation, vascular inflammation, and collagen synthesis in arterial walls. SIRT1 overexpression also decreased the association between nuclear factors on specific binding sites on TGF [69].

In addition to these studies on the whole vascular wall in hypertensive animal models, many observations on the epigenomic regulation have been done on specific cells of the vascular wall or the cells interacting with vessels such as the blood cells circulating around the body. We next outline these investigations.

### 3.2. Epigenomic Regulation in Vascular Smooth Muscle Cells (VSMCs)

Vascular smooth muscle cells (VSMCs) play an integral role in the regulation of peripheral resistance by modulating vascular tone. Autonomic nervous activity as well humoral agents are able to regulate contraction of VSMCs, leading to changes in BP [70]. It has been noted that VSMCs play a key role in the pathophysiology of hypertension due to their remarkable ability to dedifferentiate, allowing them to switch between contractile and synthetic states, in response to environmental cues or stress [71]. Studies have shown that this phenotypic switch between contractile and synthetic states in VSMCs could be governed by epigenetic modifications [71,72]. In addition, our recent studies also showed the alteration of intrinsic VSMC stiffness contributes to the development of hypertension [68,69]. A thorough understanding of epigenetic regulatory mechanisms in the VSMCs is mandatory as we develop new antihypertensive therapies.

Ten-eleven translocation-2 (TET2), a key enzyme in the DNA demethylation pathway, is a major governor of SMC plasticity and is highly expressed in VSMCs [13]. TET2 knockdown leads to a decreased expression of contractile markers such as myocardin (MYOCD), serum response factor (SRF), and myosin heavy chain 11(MYH11) and an increase in proliferative markers such as kruppel like factor 4 (KLF4) and myosin heavy chain 10 (MYH10), while TET2 overexpression restored contractile markers and inhibited synthetic genes, suggesting that TET2 serves as an important switch in VSMCs [13]. Importantly, it was shown that TET2 binds to the promoters of MYOCD, SRF, and MYH11, implicating its role in demethylating contractile genes [13]. Stimulation of the TET proteins may offer therapeutic potential in hypertension and atherosclerosis where pathological VSMC switching have been described, and re-differentiation of VSMCs is needed.

In addition to DNA methylation, histone modifications are also found to be able to modulate gene expression in VSMCs. Serum response factor (SRF), a key mediator of SMC transcription, binds to highly conserved domains near gene promoters known as the SRF binding sites or CArG boxes. Recruitment of SRF to the CArG boxes of SMC marker genes has been implicated to be associated with hyperacetylation of H3 and H4 in SMC-differentiated cells [20]. SRF complexes with cAMP-response element-binding protein (CREB)-binding protein on the hyperacetylated *SM22* gene promoter, leading to its expression [73]. Moreover, p300 mediated-acetylation of myocardin is critical for its dissociation from the inhibitory effects of histone deacetylase 5 (HDAC5) [74,75]. Myocardin’s acetylation enhances its binding of SRF and the CArG boxes and is required for VSMC gene transcription [76]. Interestingly, our recent studies showed that upregulation of SRF/myocardin in VSMCs mediates the intrinsic VSMC and aortic stiffness in hypertension [77,78], indicating the key role of this signaling pathway in the regulation of BP. The further investigation of the epigenetic regulation of SRF/myocardin signaling may be a promising target for future studies.

It is generally accepted that oxidative stress and mild chronic vascular inflammation contribute to the pathophysiology of hypertension [79]. Activation of the nucleotide-binding oligomerization domain-like receptor protein 3 (NLRP3) inflammasome, a cytosolic complex for early inflammatory responses, generates proinflammatory cytokines such as interleukin 1 β (IL-1β) and interleukin 18 (IL-18) through the activation of caspase-1 [80]. These cytokines have been implicated in hypertension and consideration should be given to their therapeutic potential [81]. Recently, the *NLRP3* gene promoter was shown to have increased acetylation at lysine 9 of histone 3 and HAT expression in SHR VSMCs [21]. Importantly, inhibition of NFκβ and HAT by curcumin prevented the activation of the NLRP3 inflammasome, VSMC phenotypic switching, and proliferation in VSMCs of SHR [21]. These findings imply that inhibition of the inflammasome, IL-1β, IL-18, and HAT by reducing histone acetylation at the *NLRP3* gene promoter may prove effective in controlling chronic inflammation in hypertension and reducing the pathology of the disease.

### 3.3. Epigenomic Regulation in Endothelial Cell Dysregulation

While VSMCs comprise a major part of the medial layer of vascular wall, the endothelial cells which line the walls are in direct contact with circulating stimuli and their contribution to the BP control has been extensively studied. It has been widely accepted that endothelial dysfunction plays an important role in systemic hypertension. Both DNA methylation and histone modification have been implicated in the regulation of endothelial cells in hypertension.

The endothelium releases various vasoactive factors, some of which are vasodilatory, e.g., nitric oxide (NO), prostacyclin (PGI_2_), and endothelium derived hyperpolarizing factor (EDHF); while some of which are vasoconstrictive, e.g., thromboxane (TXA_2_) and endothelin-1 (ET-1) [82]. The endothelial nitric oxide synthase (eNOS), which is expressed primarily in endothelial cells, is the primary mechanism by which NO is produced in the vessel. Disturbances in the NO pathway have been linked to the predominance of vasoconstrictors which feed vicious cycles to maintain high BP [83]. Notably, the expression of eNOS may be controlled by cell-specific histone modifications as acetylated histone H3 lysine 9, histone H4 lysine 12, and di- and tri-methylated lysine 4 of histone H3 are all present in the *NOS3* gene promoter in human umbilical vein endothelial cells (HUVEC) and human dermal microvascular endothelial cells (HMVEC) but are absent in non-eNOS expressing cells like VSMCs and HeLa cells [22]. This mechanism was further explained by the discovery of HDAC1 selectively bound to the *NOS3* gene promoter in VSMCs, thus reducing the histone acetylation and transcription of eNOS in SMC [22]. Epigenetic regulation of eNOS expression is key to the tissue specificity observed in NO and dysregulation of epigenetic signaling during disease may contribute to increased vascular tone as a result of decreased NO synthesis in vessels.

In addition, an experiment in the deoxycorticosterone acetate (DOCA) salt-sensitive hypertension model of the Wistar rat, showed that animals treated with resveratrol, a known antihypertensive agent, increased the total antioxidant capacity and hydrogen sulfide levels which are independent of the change NO levels in circulation [84]. It also showed that resveratrol altered the staining of the H3K27me3 pattern of the aorta and renal artery sections, suggesting that its protective effects may be due to its effects of epigenetic modifications of the vessels [84].

## 4. Clinical Application of Epigenomic Studies in Human Systemic Hypertension

Although VSMCs and ECs are the main components of the vascular wall, inflammatory cells and circulating blood cells also play essential roles in the development of hypertension, and their epigenetic regulation should be reported. As human studies increase and biomedical technology advances, many researchers have opted to use peripheral blood to isolate DNA and measure epigenetic programming. While one can speculate that there may be cell to cell or tissue to tissue variation within the human, these studies offer a powerful tool in exploring differences in epigenetic patterns from patient to patient and identifying potential risk factors and key marks. One of the first questions that researchers asked was whether there was a difference in the DNA methylation of hypertensive patients from normotensive individuals that can be detected in blood cells. Interestingly, the total 5mC level was shown lower in the peripheral blood cells of patients with essential hypertension, and even lower in patients with Stage 1 hypertension [85]. This finding indicates that some epigenomic signatures in hypertensive patients can be detected in the peripheral blood at an early stage of hypertension, thus providing a predictive biomarker for the development of hypertension. While initial DNA methylation studies focused on the global level of 5mC, subsequent research has shown that the epigenetic regulation of specific DNA sequences in peripheral blood cells can contribute to the hypertensive state.

Since hypertension is a multifactorial disease which may be mediated by alterations in multiple biological pathways, research continues to identify possible targets whose manipulation may have effects on systemic BP. It has been shown that DNA variation is correlated with DNA methylation. Genome-wide linkage and association studies (GWAS) have identified single nucleotide polymorphisms (SNPs), which influence BP. SNPs that create CpG sites may be targets for epigenetic modifications, just as loss of these sites will prevent DNA methylation. For example, a recent trans-ancestry genome-wide association study identified genetic variants at 12 new loci which have been differently DNA methylated in hypertension, four of these genes encoded proteins that participate in the regulation of vascular tone and VSMC plasticity, including: insulin like growth factor binding brotein 3 (IGFBP3), potassium two pore domain channel subfamily K member 3 (KCNK3), phosphodiesterase 3A (PDE3A), and PR domain-containing protein 6 (PRDM 6) [86]. It is remarkable that a two-fold enrichment between sentinel BP single nucleotide polymorphisms (SNPs) and DNA methylation was observed, highlighting the importance of genetic variation and providing an additional hypothesis for the racial disparity observed in the incidence of hypertension [86]. While additional studies are needed to confirm whether DNA methylation of these specific genes is enough to cause changes in BP, it is exciting to note that DNA methylation explains part of the relationship between sentinel SNPs and BP (*r* = 0.52; *p* = 0.005) [86].

In a study of the DNA methylation levels of 5 CpG dinucleotides in 62 patients, reduced promoter methylation of the α-adducin (*ADD1*) gene was been shown to be linked to an increased essential hypertension risk [14]. Females were shown to have higher methylation of the *ADD1* promoter, with a sharp decline in post-menopausal women, suggesting that estrogen signaling may also influence epigenetic regulation of the *ADD1* gene [14]. Although α-adducin is a cytoskeletal protein, an *ADD1* variant has been implicated in stimulation of renal sodium reabsorption and, subsequently, hypertension [87].

Epithelial sodium channel (ENaC), a nonvoltage-gated sodium channel, comprises three homologous subunits (α, β, and γ), with the alpha subunit (*SCNN1A*) being indispensable for channel activity [15]. The ENaC plays a critical role in maintaining extracellular fluid volume and BP. Hypermethylation of *SCNN1A* at CpG islands within the transcriptional region was associated with increased risk of developing hypertension. Importantly, the methylation level was regulated by age, gender, and antihypertensive therapy [15]. Hypermethylation of CpG islands within exons is typical of expressed genes, suggesting that *SCNN1A* gene is highly expressed in hypertension. Furthermore, CpG1 hypermethylation and CpG2 hypomethylation of the gene promoter of the amiloride-senstive sodium channel beta subunit (*SCNN1B*) were linked to hypertension, with the tested antihypertensive therapy only affecting CpG1 levels [16].

Toll-like receptors (TLRs) contribute to chronic inflammation, a mechanism that has be shown to play a role in the development of hypertension [17]. Hypomethylation of the *TLR2* promoter at CpG6 was shown to be associated with an increased risk of systemic hypertension [17]. Notably, an important link between environmental cues, such as alcohol consumption and smoking, and specific CpG islands were identified as the risk factors of hypertension [17]. The identification of methylation patterns as a result of behavior is significant since hypertension has often been described as a lifestyle disease but precise mechanisms by which chronic inflammation through TLRs contribute to hypertension are still unclear.

Euchromatic histone-lysine in methyltransferase 2 (EHMT2; also called G9a) is a lysine histone methytransferase found in leukocytes which regulates the expression of IL-17 through methylation pattern H3K9me2 [88,89]. In a longitudinal genome wide methylation study of Roux-en-Y bypass patients, the EHMT2 promoter was found to be hypomethylated in hypertension, suggesting that epigenetic regulation of EHMT2 contributes to vascular pathophysiology [18]. It remains to be explored whether IL-17 has effects in human hypertension and whether epigenetic regulation of EHMT2 is alter the hypertensive state.

Together, all of the evidence indicates that, although epigenetic regulations of systemic hypertension in the vasculature are far from fully understood, the study of epigenetic regulation in systemic hypertension holds valuable potential for the identification of biomarkers and may provide for useful therapeutic targets. The discovery of new drugs based on the epigenomic regulation of the hypertensive vasculature, both synthetic and natural, offers new hope to protect ourselves against the silent killer.

## 5. Mechanisms Underlying Epigenetic Alterations in Hypertension

It is well known that hypertension can be influenced by genetic and environmental factors. Although the mechanisms underlying the epigenetic alterations during the development of hypertension have not been completely elucidated, epigenetic programming caused by adverse fetal environments, in utero, has been shown to be strongly correlated with the hypertension in adult offspring [90]. For example, intrauterine exposure to a maternal low-protein diet in the rat results in the development of hypertension in adult offspring rats which is associated with hypomethylated *Atgr1β* gene promoters along with increased adrenal expression of AT1bR [7,91,92,93]. In addition, prenatal inflammation exposure due to the maternal inflammatory diseases is highly associated with adult hypertension in the offspring [94,95,96,97]. It has been also showed that prenatal exposure to lipopolysaccharide (LPS), a nonspecific immune-inflammatory stimulant, led to hypertension in adult offspring rats, correlating with the augmentation of histone H3 acetylation (H3AC) on the angiotensin-converting enzyme 1 (ACE1) promoter, which induced the upregulation of the (*ACE1*) expression in renal cortex tissues [98,99,100]. Importantly, prenatal anti-inflammatory treatment is able to prevent offspring from fetal programming hypertension [100]. These results together indicate that epigenetic alterations induced during the fetal development are crucial contributors to the development of hypertension.

In addition to these “congenital” effects, the epigenetic alterations can be acquired during the early period of life due to the increased vulnerability of the reprogramming cells to environmental stress, such as malnutrition, toxic chemicals, infections and mental stress. This concept is further supported by the epidemiological investigations on human population which showed that social and environmental stresses during the early period of life influence epigenetic processes that contribute to the adult race-based US health disparities in diseases including hypertension [101]. Furthermore, several lines of evidence suggest that environmentally-induced epigenomic alterations can be transmitted to subsequent generations with disease phenotypes [102]. These observations, together, indicate the interaction of the genetic and environmental factors on the epigenomic alterations in the development of hypertension. On the other hand, it has been shown that increased hemodynamic forces are able to induce DNA methylation and histone modification in VSMCs and ECs [103]. In accordance with these understandings, it is important to note that while the epigenomic alterations are a part of the mechanisms involved in blood pressure elevation, they can also be a consequence of blood pressure alterations. Additional studies are therefore needed to determine causative agents of human epigenomic manipulation and their roles in development of hypertension. 

Despite this evidence, the mechanisms by which DNA demethylation or histone modification are involved in the gene specific regulation of hypertension have not been fully elucidated. One mechanism suggests that the cell specific DNA demethylation is linked to a unique response to hormone stimulation [104]. Several studies suggest that interactions with sequence-specific DNA binding proteins and co-repressor complexes can target certain proteins to histones in a gene-specific manner [105,106]. Furthermore, other studies have shown that in addition to histone methylases, there are multiple histone demethylases, such lysine-specific demethylase 1-(LSD1), which removes mono- or di-methyl groups from H3K4 and the Jumonji C-(JmjC) domain-containing demethylases 5, which removes the tri-methylated modification and demethylates histones in a gene-specific manner by interactions between demethylases and DNA sequence specific nuclear factor complexes. Moreover, recent studies have shown that specific histone demethylases may regulate androgen-mediated transcriptional responses [107,108,109]. However, further studies are needed to confirm these mechanisms in the hypertensive individuals.

## 6. Conclusions

**Figure 1:**
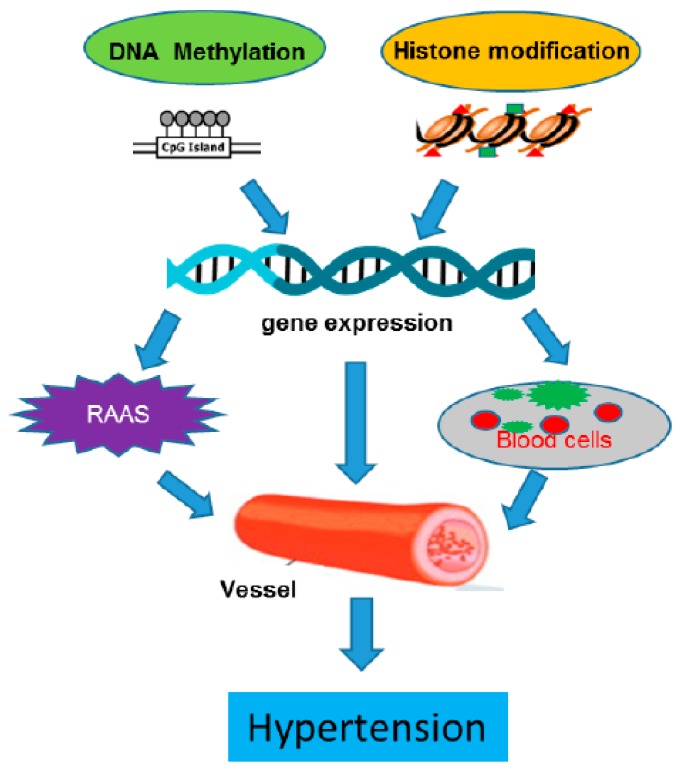
The illustration of the regulation DNA methylation and histone modification in hypertension.

The root cause of hypertension is still to be elucidated, but there is little doubt that epigenomic changes in the vessels make a contribution to the disease. As illustrated in Figure 1, epigenetic regulation participates in the development of hypertension through a comprehensive mechanism which targets different levels of complexity including the RAAS system, the vascular wall and specific cell types within the vessels. Epigenetic regulation may also affect the circulating blood cells that interact with vessels. While regulators of the RAAS system are a current key focal point, more focus should be given to the components of blood vessels themselves and their regulation.

## Figures and Tables

**Table 1:** DNA methylation and histone modification associated with hypertension.

Genes	Mark	Status	Species	Models	Tissues/Cells	Function	Ref
**DNA methylation**							
***Atgr1α***	5mC	Hypo	Rat	SHR	Aorta and mesentery artery	Increased expression of receptor and effect of RAAS	[6]
***Atgr1β***	5mC	Hypo	Rat	Maternal low protein rat		RAAS	[7]
***Ace-1***	5mC	Hypo	Mice	Maternal protein deficient mice		RAAS	[8]
***ACE-1***	5mC	Hyper	Human		Human PBMCs; cell culture (HepG2, HT29, HMEC-1, SUT)	RAAS	[9]
***HSD11B2***	5mC	Hyper	Human	Glucocorticoid treatment	Human PBMCs	Renal sodium balance	[10]
***Sslc12a2* (NKCC1)**	5mC	Hypo	Rat	SHR	Aorta and heart	Ionic balance	[11]
***ESR1* (ERα)**	5mC	Hyper	Sheep		Uterine artery	vasodilation	[12]
***SRF, MYOCD, MYH11***	5mC	Hyper	Human		Human coronary artery SMCs	contraction phenotype	[13]
***ADD1***	5mC	Hypo	Human		Human PBMCs	Ionic balance	[14]
***SCNN1A***	5mC	Hyper	Human		Human PBMCs	Ionic balance	[15]
***SCNN1B***	5mC	CpG1 Hyper, CpG2 Hypo	Human		Human PBMCs	Ionic balance	[16]
***TLR2***	5mC	Hypo	Human		Human PBMCs	Chronic inflammation	[17]
***EHMT2***	5mC	Hypo	Human		Human PBMCs	Chronic inflammation	[18]
**Histone modification**							
***Ace1***	H3Ac, H3K4me3, H3K9me2	Hyper, Hyper, Hypo	Rat	SHR	Heart, kidney	RAAS	[19]
***SM22***	H3Ac	Hyper	Mouse		10T1/2 cells	Contractile phenotype	[20]
***Nlrp3***	H3K9Ac	Hyper	Rat	SHR	VSMCs	Chronic inflammation	[21]
***NOS3*** **(eNOS)**	H3K9Ac, H4K12 H3K4 me2, H3K4me3	Hyper	Human		Cell culture; HUVEC, HMVEC, VSMC, HEPG2, HeLa, JEG-3	Vasodilation in endothelial cells	[22]
***Slc12a2* (NKCC1)**	H3Ac H3K27me3	Hyper, Hypo	Rat	Angiotensin II delivery	Aorta	Ionic balance	[23]

Abbreviations: 5mc—5-methylcytosine, RAAS—Renin-angiotensin-aldosterone system, PBMC—peripheral blood mononuclear cell, SHR—spontaneously hypertensive rats, VSMC—vascular smooth muscle cell, HUVEC—human umbilical vein endothelial cell, HMVEC—human dermal microvascular endothelial cells, H3Ac—Histone 3 acetylation, H3K4me2—dimethylation of histone 3 lysine 4, H3K4me3—trimethylation of histone 3 lysine 4.
